# “Slalom”: Microsurgical Cross-Over Decompression for Multilevel Degenerative Lumbar Stenosis

**DOI:** 10.1155/2016/9074257

**Published:** 2016-07-18

**Authors:** H. Michael Mayer, Franziska Heider

**Affiliations:** Spine Center, Schön Klinik München Harlaching, Paracelsus Medical School Salzburg, Harlachinger Straße 51, 81547 München, Germany

## Abstract

*Objective.* Selective, bilateral multisegmental microsurgical decompression of lumbar spinal canal stenosis through separate, alternating cross-over approaches.* Indications*. Two-segmental and multisegmental degenerative central and lateral lumbar spinal stenosis.* Contraindications*. None.* Surgical Technique.* Minimally invasive, muscle, and facet joint-sparing bilateral decompression of the lumbar spinal canal through 2 or more alternating microsurgical cross-over approaches from one side.* Results.* From December 2010 until December 2015 we operated on 202 patients with 2 or multisegmental stenosis (115 f; 87 m; average age 69.3 yrs, range 51–91 yrs). All patients were suffering from symptoms typical of a degenerative lumbar spinal stenosis. All patients complained about back pain; however the leg symptoms were dominant in all cases. Per decompressed segment, the average OR time was 36 min and the blood loss 45.7 cc. Patients were mobilized 6 hrs postop and hospitalization averaged 5.9 days. A total of 116/202 patients did not need submuscular drainage. 27/202 patients suffered from a complication (13.4%). Dural tears occurred in 3.5%, an epidural hematoma in 5.5%, a deep wound infection in 1.98%, and a temporary radiculopathy postop in 1.5%. Postop follow-up ranged from 12 to 24 months. There was a significant improvement of EQ 5 D, Oswestry Disability Index (ODI), VAS for Back and Leg Pain, and preoperative standing times and walking distances.

## 1. Introduction

Bilateral microsurgical so-called “cross-over decompression” through a unilateral approach has become a new minimally invasive surgical treatment option for degenerative lumbar spinal stenosis [1–7].

The main advantages of this technique are the diminished “access trauma” to the paravertebral muscles and to the facet joints. In particular the inferior facet contralateral to the approach side as well as its outer capsular surroundings can be preserved completely.

In cases of central spinal or foraminal stenosis associated with degenerative lumbar scoliosis decompression can be performed from the convex side, thus preserving the stability of the heavily loaded facet joint on the concave side [6].

These advantages usually get lost in cases with bi- or multisegmental pathologies which account for more than 50% of our own patient population in the last 17 years [3, 9, 10]. Longer skin incisions are necessary to reach 2 or more segments. The paravertebral muscles have to be retracted over a longer distance and the partial resection of the inferior and superior facets has to be performed at 2 or more segments on the same approach side. This produces higher unilateral collateral damage for muscles and joints which more or less counteracts the microsurgical philosophy of this approach.

The following paper describes a new surgical technique for selective multisegmental decompression through multiple microsurgical approaches with alternating approach sides (“Slalom” Technique).

## 2. Methods

### 2.1. Surgical Goal

The goal of this technique is to achieve multisegmental bilateral decompression through separate unilateral microsurgical approaches. The spinal canal is reached through approaches with alternating the sides (e.g., left-right-left). The rationale behind this is not only to decrease the amount of unilateral access damage but also to “balance” the trauma to the tissues on the way to the spinal canal (skin, muscles, facet joints, and lamina). Bilateral decompression of the central spinal canal and of the lateral recess is possible. If foraminal (“far lateral”) decompression has to be achieved as well, the approach has to be chosen from the contralateral side.

### 2.2. Indications

Patents with central, lateral, and foraminal stenosis of 2 and more lumbar levels with typical clinical symptoms (neurogenic claudication, buttock, leg pain, heaviness in the legs w/wo radicular symptoms, and w/wo associated deformity (e.g., degenerative lumbar scoliosis and degenerative spondylolisthesis) were included. There were no general contraindications to this approach.

### 2.3. Surgical Technique

**Figure 1:**
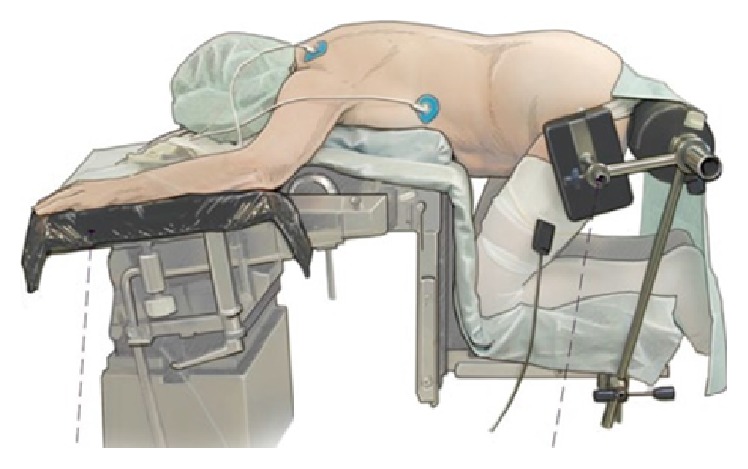
From [8], with permission. Positioning of the patient in knee-thorax position with no pressure on abdomen.

The patient is placed in a so-called knee-thorax position (Figure 1). He is kneeling on the surgical table with hips and knees flexed 90° and shoulders in 90° abduction and 90° external rotation (attention: avoid overextension of the shoulders). Elbow (N. ulnaris) and wrist joint (N. medianus) and the shins are positioned on gel pads to avoid pressure sores. The abdomen should “hang” freely to maximally lower the pressure in the epidural veins. Lateral supports are important to secure the patient while the OR table is tilted during the procedure (Figure 1).

**Figure 2:**
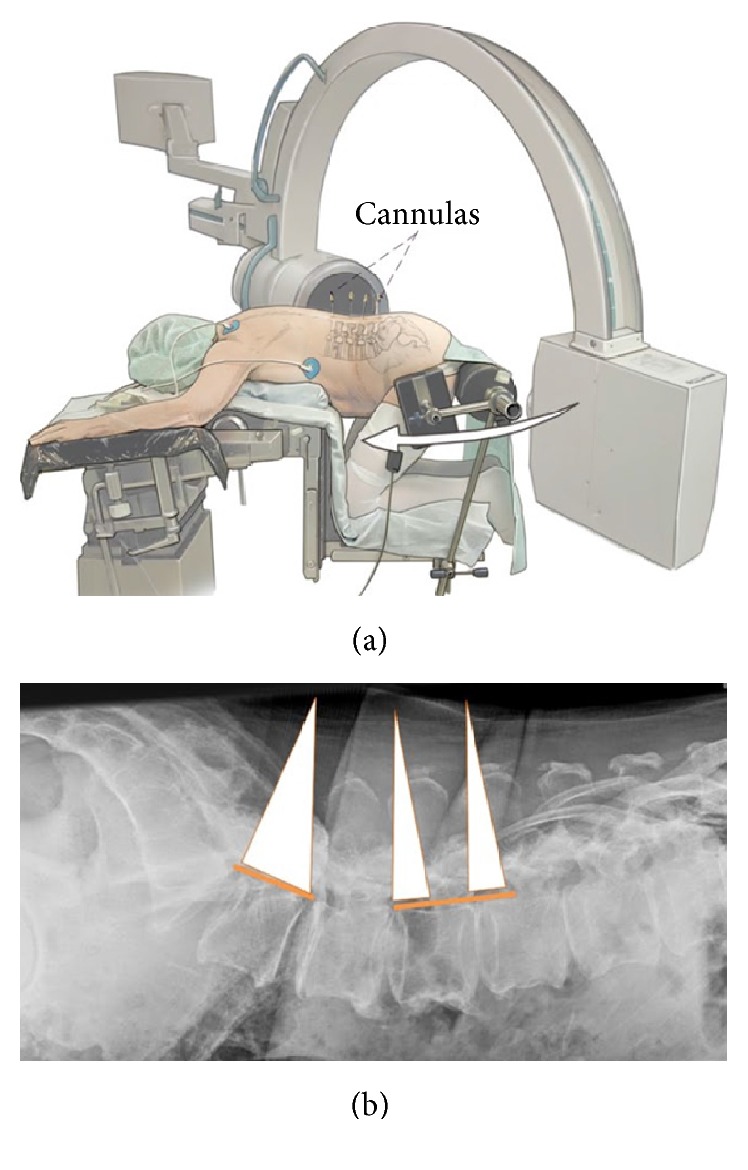
(a) From [8] with permission. Localization of the levels to be approached. Cannulas are inserted to mark the levels under fluoroscopic control. (b) From [8] with permission. Lateral X-ray with graphic marking of the approach corridors to the levels L3-4-5-S1. Due to the lordotic angle of the lumbosacral junction, the levels L4-5-1 can be approached through one small incision while the other two levels are approached through separate skin incisions (“Giant Slalom”).

**Figure 3:**
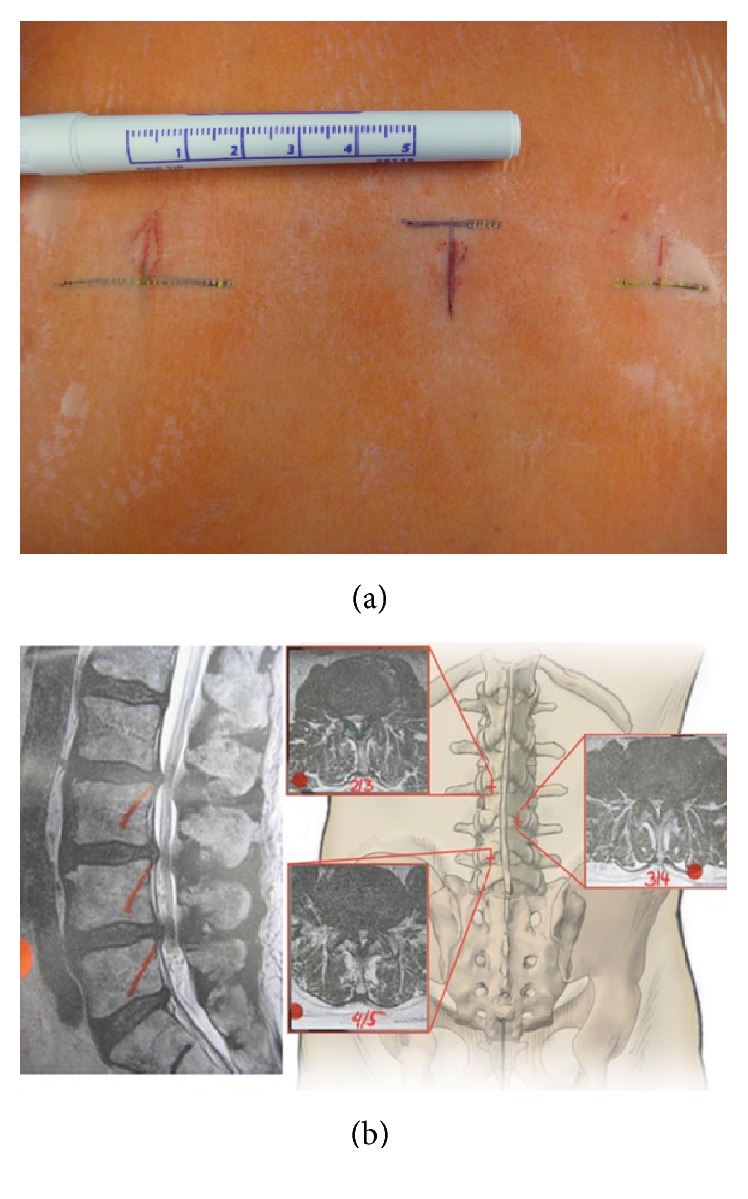
(a) From [8] with permission. Marking of the skin incisions to approach the levels L3-4-5-S1. (b) From [8] with permission. Graphic demonstration of a “Slalom” approach to stenotic levels L2-3 from the right side, L3-4 from the left, and L4-5 again from the right side.

The projection of the disc space on to the skin level is then marked under fluoroscopic control with cannulas (Figures 2(a)-2(b)). If there is a fixed lordosis sometimes 2 segments can be reached through one 20 mm skin incision.  Figure 3 shows various skin incisions to 2-3-4-5 segments (Figures 3(a)-3(b)). Surgery is performed skin-to-skin with the help of a surgical microscope (Zeiss N 700, Zeiss, Oberkochen, Germany) with variable 400 mm focus length.

**Figure 4:**
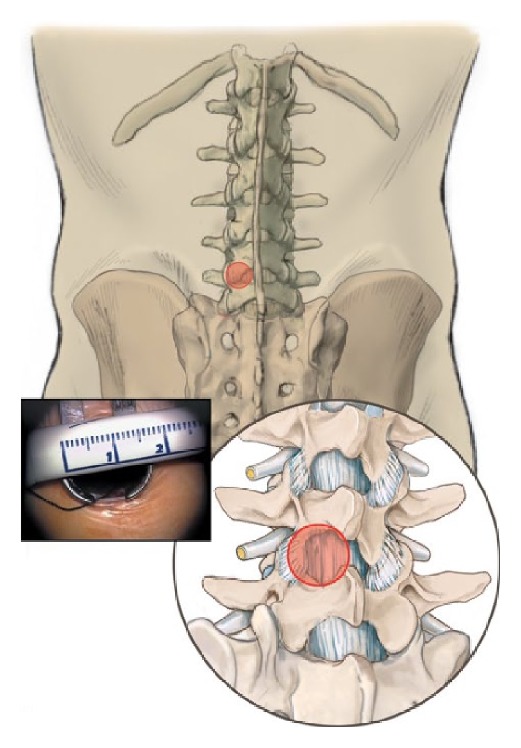
From [8] with permission. Graphic presentation of the interlaminar approach window with a minispeculum.

About 5 mm paramedian, the dorsolumbar fascia is opened and the paravertebral muscles are bluntly and gently retracted from the lamina and the interlaminar window. Care has to be taken not to incise bigger attachments of the muscles to the spinous process. Small attachments of the rotators are cut from the inferior lateral part of the superior lamina to expose the interlaminar window and the facet joint contour. A microspeculum (Piccolino, Medicon, Tuttlingen, Germany) is then inserted and the level of exposure is checked under fluoroscopic control (Figure 4).

**Figure 5:**
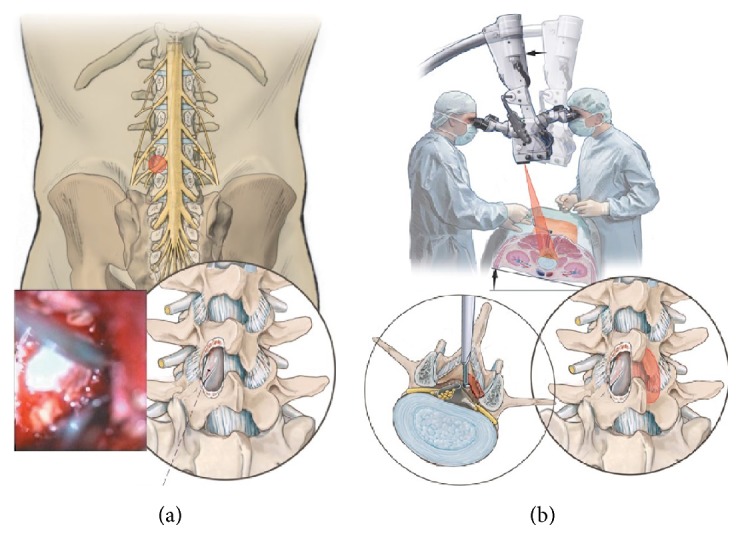
(a) From [8] with permission. Decompression of the ipsilateral side. (b) From [8] with permission. Decompression of the contralateral side.

**Figure 6:**
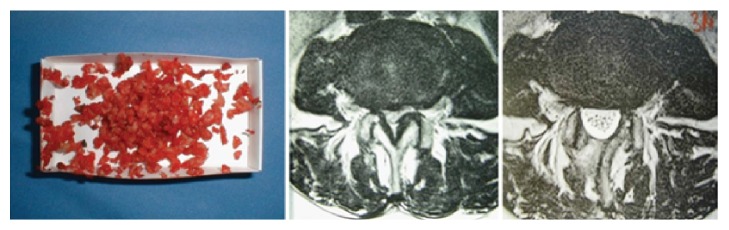
From [8] with permission. “Technical result” of decompression of one level (L3-4). Left: material that has been removed (yellow ligament, bone). Middle: MRI preoperatively; right: MRI postoperatively shows a complete decompression with preserved facet joint and minimum scar tissue formation in the muscles.

The first step of decompression is to undercut the proximal lamina with a high speed burr to expose the attachment of the yellow ligament medially and cranially. Then the spinal canal is opened and the yellow ligament is removed starting in the midline and then towards lateral cranial and finally along the lateral recess to expose the thecal sac and the root until it leaves the spinal canal around the caudal pedicle (Figure 5(a)). The cranial rim of the caudal lamina is undercut 2-3 mm. Thus the ipsilateral decompression is complete (Figure 5(a)). Now the OR table is tilted to the contralateral side and the assistant who fixes and guides the speculum tilts it to the contralateral side (Figure 5(b)). The surgical microscope is adjusted to give an oblique view to the contralateral part of the spinal canal. The yellow ligament is removed and the proximal lamina is undercut as is the superior facet on the contralateral side. Thus the central part as well as the lateral part (lateral recess) is completely decompressed as well (Figure 5(b)). Hemostasis is achieved with repeated irrigation with saline solution or the use of Floseal (Floseal Baxter Deutschland GmbH, Unterschleissheim, Germany). The table is tilted into the neutral position, the speculum is removed, and the fascia and skin are closed with resorbable intracutaneous sutures. Submuscular drainage without vacuum is inserted if necessary. A postop MRi shows the amount of decompression and the lack of “collateral damage” (Figure 6). This procedure is then repeated in the other segments with alternating skin incisions (see Figure 3).

## 3. Results

From December 2010 until December 2015 we operated on 202 patients with 2-segmental or multisegmental stenosis (115 f; 87 m; average age 69.3 yrs, range 51–91 yrs). In 202 patients a total of 577 segments were decompressed through separate approaches (see patient data listed below). All patients were suffering from symptoms typical for a degenerative lumbar spinal stenosis. All patients complained about back pain; however the leg symptoms were dominant in all cases.

Patient data are as follows:

 
*N* = 202,
 f : m (115 : 87),
 age: average 69.3 yrs (range 51–91 yrs),
 follow-up: 12–24 mos,
 Operated segments are as follows:
   2 segments (*n* = 84):
     L1-2-3 (*n* = 8),
     L2-3-4 (*n* = 14),
     L3-4-5 (*n* = 32),
     L4-5-1 (*n* = 22),
     T12-L1-2 (*n* = 3),
     L2-3 + L4-5 (*n* = 2),
     L1-2 + L3-4 (*n* = 1),
     L3-4 + L5-1 (*n* = 2),
   3 segments (*n* = 75):
     L1-2-3-4 (*n* = 16),
     L2-3-4-5 (*n* = 28),
     L3-4-5-1 (*n* = 24),
     L1-2-3 + L5-1 (*n* = 4),
     L2-3-4 + L5-1 (*n* = 3),
   4 segments (*n* = 31):
     L1-2-3-4-5 (*n* = 14),
     L2-3-4-5-1 (*n* = 11),
     L1-2-3-4 + L5-1 (*n* = 4),
     T12-L1-2-3-4 (*n* = 2),
   5 segments (*n* = 12):
     L1-2-3-4-5-1 (*n* = 11),
     T12-L1-2-3-4 + L5-1 (*n* = 1).

**Figure 7:**
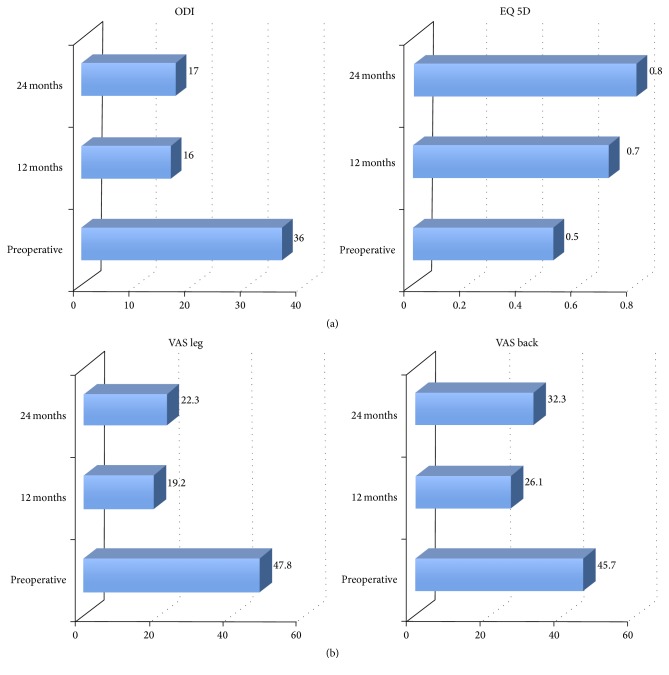
(a) EQ5 D and Oswestry Disability Index (ODI) preoperatively, as well as 12 and 24 mos postoperatively. (b) VAS for Leg and Back Pain preoperatively as well as 12 and 24 mos postoperatively.

Per decompressed segment, the average OR time was 36 min, and the blood loss 45.7 cc. Patients were mobilized 6 hrs postoperatively and hospitalization averaged 5.9 days (which was mainly due to reimbursement regulations in Germany). A total of 116/202 patients did not need submuscular drainage. All patients received a soft lumbar brace (Lumbotrain®, Fa Bauerfeind, Germany) for 4 weeks postop. Postop follow-up ranges from 12 to 24 months. There was a marked improvement of EQ 5D and Oswestry Disability Index (ODI) (Figure 7(a)). The same is true for the VAS for Back and Leg Pain (Figure 7(b)). All patients reported a significant improvement of their preop standing times and walking distances. A total of 27/202 patients suffered from a complication (13.4%). Dural tears occurred in 3.5%, epidural hematoma in 5.5%, a deep wound infection in 1.98%, and a temporary radiculopathy postop in 1.5%.

## 4. Discussion

Degenerative lumbar spinal stenosis is gaining increasing importance. Growing life expectancy with higher demands towards quality of life and better diagnostic options have made spinal stenosis the most frequent pathology seen in spine centers around the Western World.

Conventional laminectomy with removal of posterior bony and ligamentous structures has been the gold standard of surgical treatment for decades. Although postoperative development of segmental instability is a multifactorial problem, unnecessary damage to anatomic structures which stabilize the functional spinal unit has always been a problem with this technique [11–14]. Moreover, the fact that the spinal canal is exposed more than what would be necessary just for a decompression increases the contact surface between paravertebral muscles and the dura is one of the reasons for extensive scar tissue formation and epidural fibrosis following conventional laminectomy which may lead to tethering of the cauda equina and radicular symptoms [12, 15–18].

Microsurgical cross-over decompression through a unilateral approach significantly minimizes these problems [3, 4, 9, 10, 19]. The muscles are retracted only on one side and the area of the spinal canal which is exposed to the surrounding tissue remains small. This reduces the area of potential scar formation. Moreover, the integrity of the contralateral facet joint remains nearly completely intact.

In multisegmental stenosis however the sum of several unilateral interlaminar exposures leads to a more extensive unilateral muscle trauma. The removal of the medial part of the inferior facet in 2 or more levels on the same side may also lead to unilateral functional problems on the joint level. This gains even more importance in cases where spinal stenosis is associated with a degenerative deformity such as degenerative spondylolisthesis or de novo scoliosis. The “Slalom” Technique described in this paper leads to a more “balanced” collateral damage pattern thus keeping the full advantages of this minimal invasive approach.

Intraoperative blood loss was low, and submuscular drainage was necessary in only 42.5% of the cases. Minimal surgical trauma allows for early mobilization of the patient. Our success rates correspond well with those described for monosegmental approaches.

The following limitations of the Slalom approach and of our short-term experience should not remain unmentioned.

In case a dural tear occurs intraoperatively on the ipsilateral approach side, repair is only possible with the use of dura clips and patches (e.g., TachoSil®, Takeda Ltd., UK). In case of larger defects the approach has to be enlarged to perform a proper suture. This is also true for contralateral dural tears which then would require a contralateral approach.

The lordotic curvature at the levels L4-5-S1 sometimes suggests a 2-segmental decompression through one small skin incision on the same side (“Giant Slalom”). However, since this can be associated with the unilateral violation of the 2 inferior facets (L4 and L5) as mentioned above, we only recommend it in cases with wide isthmus interarticularis in order to prevent fatigue fractures postoperatively.

Our postop follow-up ranges between 1 and 2 years which may be too short for proof or disproof of a surgically induced progression of a preexisting deformity.
